# Molecular species delimitation analysis of *Leptotrombidium* spp. and other chigger species parasitizing birds in Malaysia

**DOI:** 10.1093/jme/tjaf078

**Published:** 2025-06-19

**Authors:** Praveena Rajasegaran, Kim-Kee Tan, Jing Jing Khoo, Mohammad Saiful Mansor, Mohd K S Ahmad Khusaini, Sazaly AbuBakar, Zubaidah Ya'cob, Benjamin L Makepeace

**Affiliations:** Tropical Infectious Diseases Research and Education Centre (TIDREC), Higher Institution Centre of Excellence (HICoE), Universiti Malaya, Kuala Lumpur, Malaysia; Tropical Infectious Diseases Research and Education Centre (TIDREC), Higher Institution Centre of Excellence (HICoE), Universiti Malaya, Kuala Lumpur, Malaysia; Institute of Infection, Veterinary & Ecological Sciences, University of Liverpool, Liverpool, UK; Department of Biological Sciences and Biotechnology, Faculty of Science & Technology, Universiti Kebangsaan Malaysia, Bangi, Malaysia; Wildlife Conservation Division, Department of Wildlife and National Parks Peninsular Malaysia, Ministry of Natural Resources, Environment and Climate Change, Kuala Lumpur, Malaysia; Tropical Infectious Diseases Research and Education Centre (TIDREC), Higher Institution Centre of Excellence (HICoE), Universiti Malaya, Kuala Lumpur, Malaysia; Tropical Infectious Diseases Research and Education Centre (TIDREC), Higher Institution Centre of Excellence (HICoE), Universiti Malaya, Kuala Lumpur, Malaysia; Institute of Infection, Veterinary & Ecological Sciences, University of Liverpool, Liverpool, UK

**Keywords:** barcode, bird chiggers, conspecific, cytochrome c oxidase, cryptic diversity, trombiculid mite

## Abstract

Trombiculid mites (Acariformes) are unique among arthropods of medical importance in that only the larval instar (chigger) is parasitic, which can result in the transmission of zoonotic scrub typhus. The use of molecular approaches for chigger species discrimination has been very limited until recently, especially for those parasitizing bird hosts, where data remain scarce. Here, we aimed to generate DNA barcodes of chiggers parasitizing birds in Malaysia based on the mitochondrial cytochrome *c* oxidase subunit I (COI) gene following DNA extraction, PCR and sequencing. Fifty-four COI sequences from 8 bird-associated chigger species in Malaysia were combined with 50 GenBank sequences comprising 7 genera from various countries for DNA barcode and phylogenetic analysis. The correct identification rates for the 95 COI barcodes were 96.84% (Best Match) and 86.31% (Best-Close Match). DNA barcode analyses effectively clustered the 8 nominal species from this study into their respective genera. Genetic divergence of less than 3% was observed within *Ascoschoengastia lorius*, *Neoschoengastia gallinarum*, *Parascoschoengastia heynemani*, *Leptotrombidium imphalum*, and *Blankaartia acuscutellaris*, all of which formed a monophyletic clade, confirming their conspecific nature. Conversely, intraspecific divergences of 17.64%, 15.49%, and 11.63% were obtained for *Toritrombicula densipiliata*, *Odontacarus audyi*, and *Leptotrombidium deliense*. These divergences, supported by evidence of distinct entities through delimitation analyses, indicate potential cryptic diversity within these populations. In conclusion, this study represents the first molecular genetic analysis of bird chiggers in Malaysia, revealing varying levels of genetic divergence. Our findings highlight the utility of DNA barcoding for understanding chigger diversity and aiding in accurate identification.

## Graphical Abstract

**Graphical Abstract:**
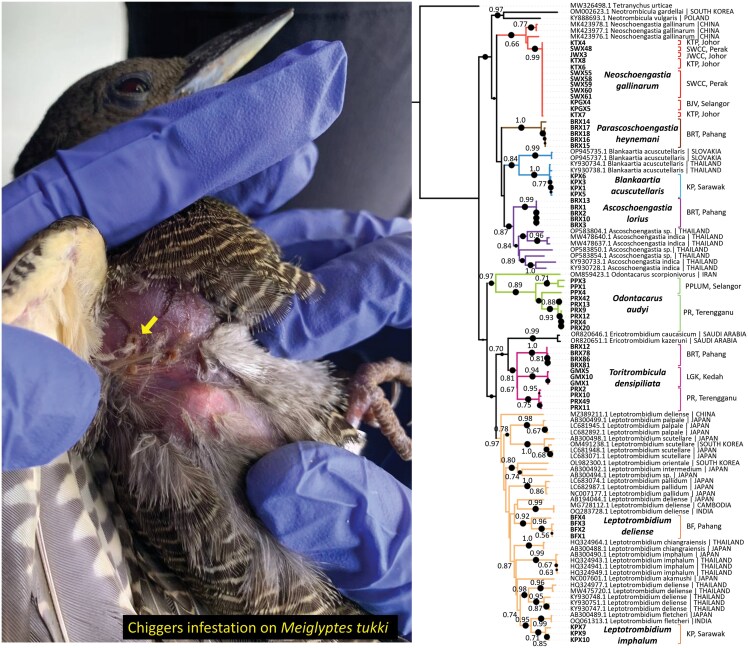

## Introduction

Larval trombiculid mites, commonly known as “chiggers” (Actinotrichida: Trombiculidae), have been closely studied since the 1940s. This increased interest was prompted by the discovery that certain chigger species were associated with a widespread zoonosis, scrub typhus, highlighting their public health importance (Fernandes and Kulkarni 2003). Chiggers act as both vectors and main reservoirs for the Gram-negative obligate intracellular bacterium *Orientia tsutsugamushi* (Hayashi, 1920), which can lead to scrub typhus disease upon infection of a human host (Pham et al. 2001). Additionally, their parasitism can cause severe dermatitis, often referred to as scrub-itch (in humans), or more broadly as trombiculiasis in a variety of vertebrate hosts (Stekolnikov et al. 2016, Molin et al. 2020, Chaisiri et al. 2023). The life cycle of trombiculid mites consists of 7 stages (Shatrov and Kudryashova 2006). Unique among arthropods of medical importance, only the larval stage of trombiculid mites is parasitic. In contrast, the postlarval instars are predators of other soft-bodied arthropods or their eggs (Dong et al. 2018, Chaisiri et al. 2023) and are difficult to identify due to high interspecific resemblance among adults (Lee and Choi 2022). Consequently, the primary focus of research, as well as taxonomic emphasis, is placed on chiggers, the larvae (Makol et al. 2010).

Chigger identification primarily relies on morphological identification techniques, which offer valuable insights into taxonomy but are challenging to implement due to the larvae’s minute size, high level of diversity within the Trombiculidae family, and the need to source locally relevant keys (Elliott et al. 2019). According to Nielsen et al. (2021), there is ongoing uncertainty regarding the identification of chiggers, as highlighted in several taxonomic monographs (eg Domrow and Lester 1985, Wen 2004, Kudryashova and Stekolnikov 2010, Stekolnikov 2013). The difficulties of stabilizing the taxonomy of chigger mites have been evident through various instances of misidentifications, proposed replacement names, and *lapsus calami* (Nielsen et al. 2021). Despite efforts to address these challenges, confusion persists in classifying certain species, subfamilies, and genera. Stekolnikov et al. (2021) expressed the persisting uncertainty regarding the actual number of described trombiculid species, despite the publication of detailed local reviews of chigger fauna in recent decades. While Brennan and Goff (1977) estimated the number of chigger species to be approaching 3,000, Zhang et al. (2011) suggested a count of 3,628 species, including 230 species of Leeuwenhoekiidae, 3,100 species of Trombiculidae (*sensu stricto*), and 298 species of Walchiidae (now known as Gahrliepiinae). However, Nielsen et al. (2021) presented a checklist documenting 3,013 valid species names. In this paper, we adopt the classification framework of Stekolnikov (2021), which defines the family Trombiculidae (Ewing, 1944) as comprising 3 subfamilies: Gahrliepiinae (Womersley, 1952), Trombiculinae (Ewing, 1929), and Leeuwenhoekiinae (Womersley, 1944) ensuring the alignment with recent taxonomic reviews. Among the diverse habitats of Southeast Asia, Malaysia stands out for its rich trombiculid diversity, with 203 chigger species recorded, followed closely by Thailand with 156 species (Stekolnikov 2021, Koosakulnirand et al. 2023).

Recent advancements in identification techniques, such as autofluorescence visualization and genetic barcoding, have greatly enhanced the potential for improved taxonomic clarity (Kumlert et al. 2018). However, common challenges persist in chigger taxonomy, including a limited understanding of intraspecific variation, reliance on species diagnosis using insufficient material, and the use of identification keys based on specimens from limited geographic areas (Zajkowska et al. 2023). DNA barcoding has emerged as a powerful molecular identification tool across various animal groups (Hajibabaei et al. 2006, 2007, Kerr et al. 2007, Pramual et al. 2011, Kumlert et al. 2018; Yin et al. 2022), gaining recognition for its reliability in taxonomic studies, and proven effectiveness in revealing intraspecific diversity and potential cryptic species of trombiculid mites in recent years (Lee and Choi 2022, Ogawa et al. 2023, Rajasegaran et al. 2024).

A review by Antonovskaia (2018) noted that early species identification of mites relied on various DNA markers, including mitochondrial genes such as 16S and 12S ribosomal RNA, transfer RNA-Val, cytochrome oxidase subunit I (COI), and cytochrome b (Cytb), as well as nuclear loci such as 28S ribosomal RNA (28S rDNA), internal transcribed spacer 1 and 2 (ITS1, 2), and fragments of the gene encoding elongation factor 1 α (EF1α) (Cruickshank 2002; Dabert 2006). Among these, the mitochondrial gene encoding COI is the most frequently used marker for taxonomic identification of chiggers and for inferring intra- and interspecies relatedness (Young et al. 2012, Moniuszko et al. 2017, 2018, Lee and Choi 2022, Zajkowska and Mąkol 2022). In a recent study, Rajasegaran et al. (2024) found that the COI gene provided the highest resolution in distinguishing chigger specimens, with no shared haplotypes between the countries studied (Thailand, Malaysia, and China), while ITS2 and the 18S rRNA gene showed shared haplotypes. Ogawa et al. (2023) demonstrated that DNA barcoding with the COI gene uncovered broad genetic diversity within *Leptotrombidium* spp. Their redesigned COI primers exhibited higher detection sensitivity compared to the universal COI primers published by Folmer et al. (1994). They observed significant differences in the COI genes of *Leptotrombidium* mites compared to Trombiculidae of different genera (*Walchia*, *Neoschoengastia*, and *Neotrombicula*), with variations evident within species from Japan. Additionally, genetic variation was detected in *Leptotrombidium* spp. in Korea, revealing 2 haplotypes for *L. pallidum* (Nagayo, Miyagawa, Mitamura & Tamiya, 1916) and *L. palpale* (Nagayo, Miyagawa, Mitamura & Tamiya, 1919), and 3 haplotypes in *L. scutellare* (Nagayo, Miyagawa, Mitamura & Tamiya, 1916), indicating significant levels of cryptic diversity (Lee and Choi 2022). In Southeast Asia, Kumlert et al. (2018) successfully provided paired morphotype and genotype data for individual chiggers using autofluorescence and bright-field microscopy, along with a DNA barcode approach targeting the COI gene. Their study demonstrated the grouping of individual specimens based on morphotyped species. However, the availability of genetic data for trombiculid mites to date shows poor geographic and taxonomic representation.

Understanding the interaction between hosts, pathogens, and vectors, including their genomic information, is crucial for effectively managing and preventing vector-borne diseases (Kim et al. 2020). While progress has been made in regions such as Thailand (Kumlert et al. 2018), Japan (Shao et al. 2005, Ogawa et al. 2023), China (Zhou et al. 2020, Tao et al. 2022), Poland (Moniuszko et al. 2015), the Balkan region (Zajkowska et al. 2023), Brazil (Jacinavicius et al. 2018), and Saudi Arabia (Alkathiry et al. 2024), there is a notable absence of genetic data for trombiculid mites from Malaysia. DNA barcodes for chiggers, especially those specific to bird hosts in Malaysia, are currently unavailable, with the exception of a single species, *Neoschoengastia gallinarum* (Hatori, 1920), which has been identified as a potential species complex upon comparison with a population from Thailand (Rajasegaran et al. 2024). Therefore, this study aimed to obtain COI barcode sequences of trombiculids associated with birds and assess the suitability of those sequences in differentiating species of Trombiculidae in Malaysia.

## Materials and Methods

### Bird Trapping and Sample Collection

**Fig. 1.**
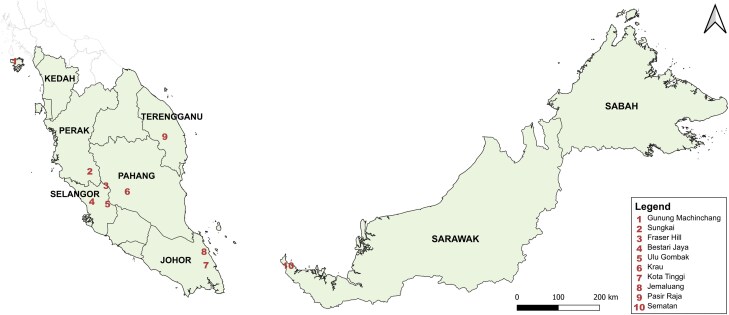
Map illustrating the 10 bird host sampling locations across Peninsular Malaysia (*n* = 9 sites) and Sarawak, East Malaysia (*n* = 1 site). 1. Gunung Machinchang Langkawi Island, Kedah (LGK—N6.429010, E99.729852); 2. Sungkai Wildlife Conservation Centre, Perak (SWCC—N4.036067, E101.366133); 3. Fraser Hill, Pahang (BF—N3.725770, E101.718333); 4. Bestari Jaya Village, Selangor (BJV—N3.378008, E101.410224); 5. Ulu Gombak Forest Reserve, Selangor (PPLUM—N3.325739, E101.752235); 6. Krau Wildlife Reserve, Pahang (BRT—N3.597957, E102.182874); 7. Kota Tinggi Plantation, Johor (KTP—N1.997487, E103.896027); 8. Jemaluang Wildlife Conservation Centre, Johor (JWCC—N2.291356, E103.852966); 9. Pasir Raja Forest Reserve, Terengganu (PR—N4.790575, E102.999348); 10. Pueh Village, Sematan, Sarawak (KP—N1.831126, E109.708966).

Field samplings for chigger collection from bird hosts were conducted at 10 sites across Peninsular Malaysia and Sarawak, Malaysian Borneo, from January 2021 to June 2022 (Fig. 1; Koosakulnirand et al. 2023). Bird trappings were conducted with expert assistance from the Department of Wildlife and National Parks (DWNP) Peninsular Malaysia. Forest passerines were trapped using 30 mist nets (8 m × 12 m; 3 mm mesh size). Ground-nesting birds, including free-range domestic chickens (*Gallus gallus domesticus*), were obtained from villages, and pheasants (*Polyplectron malacense*) were obtained using large scoop nets from sanctuaries protected by the DWNP (Rajasegaran et al. 2024). Captured birds underwent morphological identification following Robson (2008) and were visually examined for the presence of chiggers. Chiggers attached to the birds’ skin were carefully removed using forceps and lancets to minimize injury to the birds and damage to the chiggers. The recovered chiggers were preserved in 70% ethanol, individually labeled with a number/site code corresponding to each bird host and sampling site, and stored at −20 °C.

### Morphological Identification of Chiggers

The taxonomic description for each species used in this study was reported in Koosakulnirand et al. (2023). Chiggers from each host were counted, and approximately 10% were selected for permanent mounting in Berlese fluid-gum chloral (TCS Bioscience Ltd, Buckingham, UK) to remove all internal tissues for species-level identification. Mites were examined and photographed using an Axio Imager M2 microscope (Zeiss, Oberkochen, Germany) and ZEN 2011 imaging software (Kumlert et al. 2018). These individuals were not used for DNA extraction but were retained as voucher specimens and deposited at the Tick Cell Biobank Asia Outposts Laboratory, Tropical Infectious Diseases Research & Education Centre (TIDREC), Universiti Malaya (Koosakulnirand et al. 2023).

The remaining chiggers (*n* = 50) subjected to genomic DNA extraction in this study were identified at the species level using the autofluorescence microscopy method described by Kumlert et al. (2018). Morphological measurements for species identification were performed on a GXM-L3201 LED research fluorescence trinocular microscope (GT Vision LTD) equipped with GXCapture-T software (Alkathiry et al. 2024). Species identification was guided by comparisons with aforementioned voucher specimens (Koosakulnirand et al. 2023) and detailed taxonomic keys and descriptions provided in Nadchatram and Dohany (1974), Nadchatram (1963, 1967a, b), Nadchatram and Traub (1971), Vercammen-Grandjean and Langston (1976), Tanskul and Linthicum (1999), Shaw (2010), Stekolnikov (2013), Makol and Korniluk (2017), and Kumlert et al. (2018).

### DNA Extraction from Chiggers

Following species-level identification, genomic DNA was extracted from the 50 individual chigger mites using a Qiagen DNA Micro extraction kit (Qiagen, Redwood City, CA, USA) according to the manufacturer’s protocol. Individual chiggers were washed in nuclease-free water to remove ethanol. Each chigger sample was then digested in 180 µl tissue lysis buffer with 20 µl proteinase K and incubated at 56 °C overnight. Following the manufacturer’s instructions, DNA was eluted in 30 µl elution buffer and stored at −20 °C.

### PCR Amplification and Sequencing

The extracted genomic DNA was subjected to polymerase chain reaction (PCR) amplifications targeting the COI locus (forward—LCO1490: 5′-GGTCAACAAATCATAAAGATATTGG-3′; reverse—HCO2198: 5′-TAAACTTCAGGGTGACCAAAAAATCA-3′) (Folmer et al. 1994). The PCR amplification took place in 25-µl reaction volumes constituting 2 µl DNA template, 12.5 µl 2× Green DreamTaq Buffer (Thermo Scientific, Waltham, MA, USA), and 1 µl each primer (10 mM) in a 96-well SimpliAmp Thermal Cycler (Applied Biosystems, Inc., Foster City, CA, USA). The thermocycling profile was as follows: predenaturation at 95 °C (2 min), followed by 35 cycles of 95 °C (1 min) for denaturation; 40 °C (1 min) for annealing; 72 °C (1 min and 30 s) for extension; and a final extension at 72 °C (7 min). The amplified PCR products were electrophoresed on 1.0% agarose gel, prestained with SYBR Safe DNA gel stain (Invitrogen, USA) and run with a 100 bp DNA ladder (GeneDireX, Inc., Taiwan) to determine the amplicon size. The successful PCR amplicons were sent to Apical Scientific Sdn. Bhd. (Selangor, Malaysia) for purification and Sanger sequencing.

The Nucleotide Basic Local Alignment Search Tool with BLASTn online platform (https://blast.ncbi.nlm.nih.gov) was used to compare each obtained DNA sequence to the nucleotide collection database. Both forward and reverse sequences of COI were later analyzed and aligned using the ClustalW Multiple alignment (Thompson et al. 1997) program implemented in BioEdit v7.2.5 (Hall 1999) and trimmed to 567 bp. The presence of *numts* in sequences was assessed using EMBOSS: transeq, a bioinformatics tool for sequence translation, to identify premature stop codons and prevent erroneous phylogenetic conclusions (Cruickshank 2002). The 50 newly generated sequences of COI were deposited in the National Center of Biotechnology Information (NCBI) GenBank database under the accession numbers PP739731 to PP739779, and PP741800 (Table 1).

**Table 1.** Species and collection details of 8 chigger species from Malaysian bird hosts subjected to barcoding using the COI gene

Subfamily and tribe	Species	Sample ID	Host species	Host order (family)	Migratory Status	Site ID	GenBank accession no.
Leeuwenhoekiinae	*Odontacarus audyi*	PPX1	*Cacomantis sepulcralis* (Raffles, 1822)	Cuculiformes (Cuculidae)	Resident	PPLUM	PP739770
		PPX3	*Copsychus malabaricus* (Scopoli, 1786)	Passeriformes (Muscicapidae)	Resident	PPLUM	PP739771
		PPX4	*C. malabaricus*	Passeriformes (Muscicapidae)	Resident	PPLUM	PP739772
		PRX4	*Philentoma pyrhoptera* (Temminck, 1835)	Passeriformes (Vangidae)	Resident	PR	PP739774
		PRX9	*Larvivora cyane* (Pallas, 1811)	Passeriformes (Muscicapidae)	Migrant	PR	PP739775
		PRX12	*L. cyane*	Passeriformes (Muscicapidae)	Migrant	PR	PP739736
		PRX13	*L. cyane*	Passeriformes (Muscicapidae)	Migrant	PR	PP739737
		PRX20	*P. pyrhoptera*	Passeriformes (Vangidae)	Resident	PR	PP739738
		PRX42	*Stachyris poliocephala* (Temminck, 1836)	Passeriformes (Timaliidae)	Resident	PR	PP739739
Trombiculinae: Schoengastiini	*Ascoschoengastia lorius*	BRX1	*Meiglyptes tukki* (R.P. Lesson, 1839)	Piciformes (Picidae)	Resident	BRT	PP739763
		BRX2	*M. tukki*	Piciformes (Picidae)	Resident	BRT	PP739764
		BRX3	*M. tukki*	Piciformes (Picidae)	Resident	BRT	PP739765
		BRX10	*M. tukki*	Piciformes (Picidae)	Resident	BRT	PP739766
		BRX13	*M. tukki*	Piciformes (Picidae)	Resident	BRT	PP739757
	*Neoschoengastia gallinarum*	KPGX4	*Gallus gallus* (Linnaeus, 1758)	Galliformes (Phasianidae)	Resident	BJV	PP739776
		KTX4	*G. gallus*	Galliformes (Phasianidae)	Resident	KTP	PP739732
		KTX6	*G. gallus*	Galliformes (Phasianidae)	Resident	KTP	PP739731
		SWX48	*Polyplectron malacense* (Scopoli, 1786)	Galliformes (Phasianidae)	Resident	SWCC	PP739747
		SWX55	*P. malacense*	Galliformes (Phasianidae)	Resident	SWCC	PP739748
		SWX58	*P. malacense*	Galliformes (Phasianidae)	Resident	SWCC	PP739749
		SWX59	*P. malacense*	Galliformes (Phasianidae)	Resident	SWCC	PP739750
		SWX60	*P. malacense*	Galliformes (Phasianidae)	Resident	SWCC	PP739751
		SWX61	*P. malacense*	Galliformes (Phasianidae)	Resident	SWCC	PP739752
	*Parascoschoengastia heynemani*	BRX14	*Alcedo peninsulae* Laubmann, 1941	Coraciiformes (Alcedinidae)	Resident	BRT	PP739758
		BRX15	*A. peninsulae*	Coraciiformes (Alcedinidae)	Resident	BRT	PP739759
		BRX16	*A. peninsulae*	Coraciiformes (Alcedinidae)	Resident	BRT	PP739760
		BRX17	*A. peninsulae*	Coraciiformes (Alcedinidae)	Resident	BRT	PP739761
		BRX18	*A. peninsulae*	Coraciiformes (Alcedinidae)	Resident	BRT	PP739762
Trombiculinae: Trombiculini	*Blankaartia acuscutellaris*	KPX1	*Lewinia striata* (Linnaeus, 1766)	Gruiformes (Rallidae)	Resident	KP	PP739746
		KPX3	*L. striata*	Gruiformes (Rallidae)	Resident	KP	PP739745
		KPX5	*L. striata*	Gruiformes (Rallidae)	Resident	KP	PP739744
		KPX6	*L. striata*	Gruiformes (Rallidae)	Resident	KP	PP739743
	*Leptotrombidium deliense*	BFX1	*Pellorneum ruficeps* Swainson, 1832	Passeriformes (Pellorneidae)	Resident	BF	PP739753
		BFX2	*P. ruficeps*	Passeriformes (Pellorneidae)	Resident	BF	PP739754
		BFX3	*P. ruficeps*	Passeriformes (Pellorneidae)	Resident	BF	PP739755
		BFX4	*P. ruficeps*	Passeriformes (Pellorneidae)	Resident	BF	PP741800
	*L. imphalum*	KPX7	*L. striata*	Gruiformes (Rallidae)	Resident	KP	PP739742
		KPX9	*L. striata*	Gruiformes (Rallidae)	Resident	KP	PP739741
		KPX10	*L. striata*	Gruiformes (Rallidae)	Resident	KP	PP739740
	*Toritrombicula densipiliata*	BRX12	*Turdinus macrodactylus* (Strickland, 1844)	Passeriformes (Pellorneidae)	Resident	BRT	PP739756
		BRX78	*Cyanoderma erythropterum* (Blyth, 1842)	Passeriformes (Timaliidae)	Resident	BRT	PP739767
		BRX81	*Ficedula dumetoria* (Wallace, 1864)	Passeriformes (Muscicapidae)	Resident	BRT	PP739768
		BRX86	*Stachyris maculata* (Temminck, 1836)	Passeriformes (Timaliidae)	Resident	BRT	PP739769
		GMX1	*Cyornis sumatrensis* (Raffles, 1822)	Passeriformes (Muscicapidae)	Resident	LGK	PP739777
		GMX5	*C. sumatrensis*	Passeriformes (Muscicapidae)	Resident	LGK	PP739778
		GMX10	*L. cyane*	Passeriformes (Muscicapidae)	Migrant	LGK	PP739779
		PRX2	*L. cyane*	Passeriformes (Muscicapidae)	Migrant	PR	PP739773
		PRX10	*S. poliocephala*	Passeriformes Timaliidae	Resident	PR	PP739734
		PRX11	*S. poliocephala*	Passeriformes Timaliidae	Resident	PR	PP739735
		PRX49	*Cyornis brunneatus* (H.H. Slater, 1888)	Passeriformes (Muscicapidae)	Migrant	PR	PP739775

### Phylogenetic Reconstruction

The MEGA software (version 11.0.11) (MEGA11) (Tamura et al. 2021) was used to run Modeltest to estimate the best evolutionary model of nucleotide substitution for the COI sequences. The General Time Reversible (GTR) with gamma (G) distribution and evolutionary invariable (+I) substitution model was suggested as the optimal model rates showed the lowest Bayesian Information Criterion (BIC) and was chosen to best describe the substitution pattern in the rest of the phylogenetic analysis. Neighbour-joining (NJ) tree analysis was performed using Kimura’s 2-parameter substitution model inferred in MEGA11 (Tamura et al. 2021) with 1,000 bootstrap replicates. A Maximum Likelihood (ML) phylogenetic tree was constructed using online phylogeny software, PhyML 3.0, with automated model selection using BIC (Guindon et al. 2010). A total of 54 publicly available sequences comprising 7 genera from various regions were obtained from GenBank for comparative analysis. Reference sequences are detailed in Table 2, and the mitochondrial COI sequence of *Tetranychus urticae* C. L. Koch, 1836 (Trombidiformes) (MW326498.1) served as an outgroup for tree construction. Trees were visualized and edited in the Interactive Tree of Life (iTOL) (Letunic and Bork 2021).

**Table 2.** List of chigger species retrieved from GenBank subjected to barcoding comparison using the COI gene

Subfamily and tribe	Species	GenBank accession no.	Origin	References
Leeuwenhoekiinae	*Odontacarus scorpionivorus* Stekolnikov & Saboori, 2019	OM859423.1	Iran	Khadem-Safdarkhani et al. (unpublished)
Trombiculinae: Schoengastiini	*Ascoschoengastia sp.*	OP583850.1	Thailand	Linsuwanon et al. (unpublished)
		OP583854.1		
		OP583804.1		
	*Ascoschoengastia indica*	MW478637.1	Thailand	Wulandhari et al. (2021)
		MW478640.1		
		KY930728.1		Kumlert et al. (2018)
		KY930733.1		
	*Neoschoengastia gallinarum*	MK423976.1	China	Zhou et al. (2020)
		MK423977.1		
		MK423978.1		
		OR632301	Malaysia	Rajasegaran et al. (2024)
		OR632292		
		OR632305		
		OR632306		
Trombiculinae: Trombiculini	*Blankaartia acuscutellaris*	OP945735.1	Slovakia	Trnka et al. (2023)
		OP945737.1		
		KY930738.1	Thailand	Kumlert et al. (2018)
		KY930734.1		
	*Ericotrombidium kazeruni* Vercammen-Grandjean & Langston, 1976	OR820651.1	Saudi Arabia	Alkathiry et al. (2024)
	*E. caucasicum* Schluger, 1961	OR820646.1	Saudi Arabia	Alkathiry et al. (2024)
	*Leptotrombidium akamushi* (Brumpt, 1910)	NC007601.1	Japan	Mitani et al. (unpublished)
	*L. imphalum*	AB300490.1	Japan	Mitani et al. (unpublished)
		HQ324949.1	Thailand	Takhampunya et al. (unpublished)
		HQ324941.1		
		HQ324943.1		
	*L. pallidum*	LC683074.1	Japan	Ogawa et al. (unpublished)
		LC682987.1		
		NC007177.1		Shao et al. (2005)
	*L. intermedium* (Nagoya, Mitamura & Tamiya, 1920)	AB300492.1	Japan	Mitani et al. (unpublished)
	*Leptotrombidium sp.*	AB300494.1	Japan	Mitani et al. (unpublished)
	*L. orientale* (Schluger, 1948)	OL982300.1	South Korea	Lee and Choi (2022)
	*L. palpale* Wang, 1964	AB300499.1	Japan	Mitani et al. (unpublished)
		LC681945.1	Japan	Ogawa et al. (2023)
		LC682892.1		
	*L. chiangraiensis* Tanskul & Linthicum, 1997	AB300488.1	Japan	Mitani et al. (unpublished)
		HQ324964.1	Thailand	Takhampunya et al. (unpublished)
	*L. scutellare*	OM491238.1	South Korea	Lee and Choi (2022)
		AB300498.1	Japan	Mitani et al. (unpublished)
		LC683071.1		Ogawa et al. (unpublished)
		LC681948.1		Ogawa et al. (2023)
	*L. fletcheri*	AB300489.1	Japan	Mitani et al. (unpublished)
		OQ061313.1	India	Rosangkima et al. (unpublished)
	*L. deliense*	AB194044.1	Japan	Mitani et al. (unpublished)
		OQ283728.1	India	Rosangkima et al. (unpublished)
		MZ389211.1	China	Gu (unpublished)
		MW475720.1	Thailand	Wulandhari et al. (2021)
		KY930747.1	Thailand	Kumlert et al. (2018)
		KY930748.1		
		KY930751.1		
		MG728112.1	Cambodia	
		HQ324977.1	Thailand	Takhampunya et al. (unpublished)
	*Neotrombicula gardellai* Schluger, 1966	OM002623.1	South Korea	Lee and Choi (2022)
	*N. vulgaris* Schluger, 1955	KY888693.1	Poland	Moniuszko et al. (2017)

### Pairwise Genetic Distance

Pairwise genetic distance within and among species was computed using the Kimura 2-parameter (K2P) model (Kimura 2005) with 1,000 bootstrap replicates implemented in MEGA11 (Tamura et al. 2021). The effectiveness of COI sequences for species identification was assessed through the “Best Match” (BM) and “Best Close Match” (BCM) methods in the TaxonDNA software (Meier et al. 2006). The assessment was conducted using the default threshold value of 3.00% in TaxonDNA software. Ambiguous and incorrect identifications arose when utilizing single COI barcodes as queries for species. This occurred because there were no additional conspecific reference sequences in the dataset to facilitate accurate matching for these individual COI barcodes (Ross et al. 2008). Therefore, 9 single COI sequences obtained from GenBank (OM859423.1, OR820646.1, OR820651.1, NC007601.1, AB300492.1, AB300494.1, OL982300.1, OM002623.1, KY888693.1) were excluded, leaving 95 COI sequences subjected to the BM and BCM analyses.

### Species Delimitation Analyses

Species delimitation analyses involved classifying COI sequences into entities that represent putative species, typically denoted as operational taxonomic units (OTUs), and were performed using assemble species by automatic partitioning (ASAP) (Puillandre et al. 2021), Poisson Tree Processes (PTP) (Zhang et al. 2013), and Generalized Mixed Yule Coalescent (GMYC) (Pons et al. 2006, Fujisawa and Barraclough 2013) methods. These methods encompass hierarchical clustering algorithms based on both distance- and tree-based approaches (Puillandre et al. 2021, Guo and Kong 2022). The ASAP was performed on a web-based server (ASAP: https://bioinfo.mnhn.fr/abi/public/asap, accessed on 15 January 2024); using a Kimura (K80) model with default settings, TS/TV model 2.0 (Puillandre et al. 2021, Goulpeau et al. 2022). The PTP analysis was conducted on the bPTP web server (https://species.h-its.org/ptp/, accessed on 15 January 2024) using the ML tree generated from the RAxML analysis as the input tree. The analysis was run for 100,000 Markov Chain Monte Carlo (MCMC) generations with the other settings set as default. The delimitation results were based on the maximum likelihood partition (PTP_ML). To initiate the GMYC species delimitation method for the COI dataset, an ultrametric tree was generated using BEAST v2.6.6 (Bouckaert et al. 2019) and run on the CIPRES Science Gateway v3.3 online portal (https://www.phylo.org/) (Miller et al. 2010). Preceding this, an XML input file was created using BEAUti v2.6.6 (Bouckaert et al. 2019) with the best-fitting model, namely the GTR + G + I substitution, as determined by jModelTest2 (Darriba et al. 2012). The MCMC chains were run for 30 million generations, with topologies and parameters logged every 1,000 generations. The analysis was then confirmed using Tracer v1.7.1 (Rambaut et al. 2018) for an effective sampling size of more than 200, indicating that the MCMC chains had adequately converged (Drummond and Rambaut 2007). The output tree was analyzed in TreeAnnotator 2.6.6 (Bouckaert et al. 2019), discarding the initial 10% as burn-in. The subsequent GMYC analysis for the COI dataset was conducted in RStudio (RStudio Team 2020) using R packages including “ape” (Paradis and Schliep 2019), “paran” (Dinno and Dinno 2018), “rncl” (Michonneau et al. 2016), and “splits” (Ezard et al. 2009).

## Results

### Host and Chigger Sampling

**Fig. 2.**
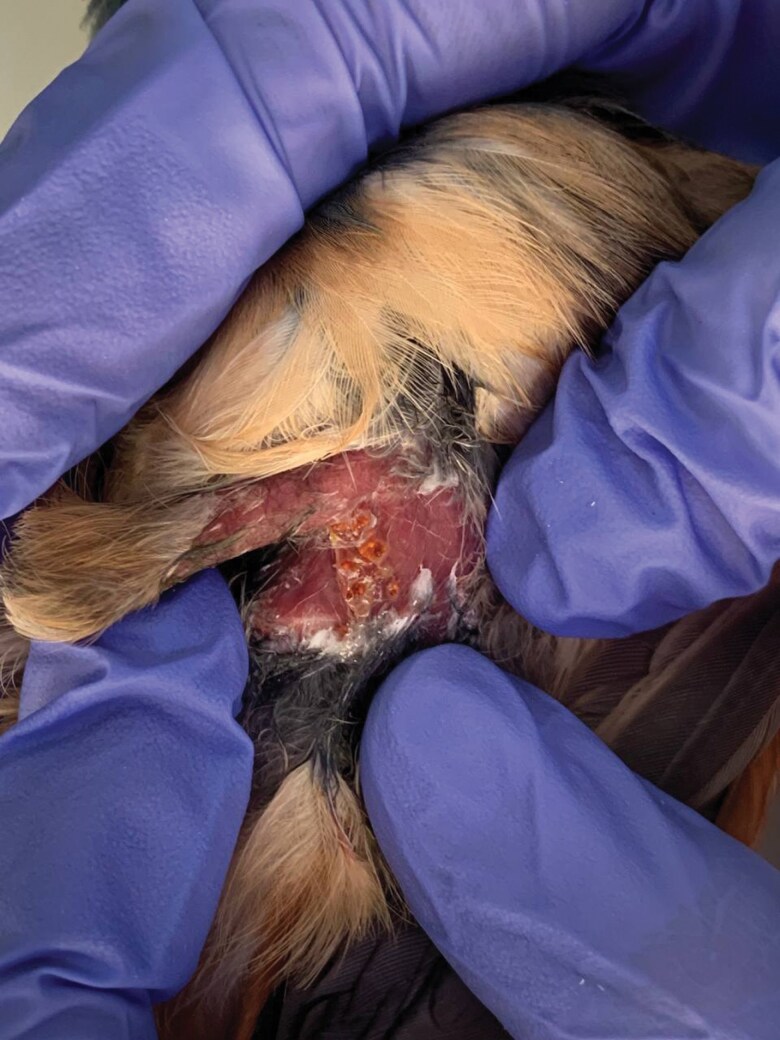
Chigger infestation on the dermal surface of the medial part of the left leg of a rufous-winged philentoma (*Philentoma pyrhoptera*).

A total of 17 bird species from both Peninsular and East Malaysia were captured and found to host chiggers. Chiggers were recovered from various body parts of the hosts, including beneath the wings, around the breast area, and within the inguinal region. Figure 2 shows chigger infestation at the medial part of the left leg of a rufous-winged philentoma (*Philentoma pyrhoptera*). The status of each bird host and its associated chiggers is presented in Table 1. Notably, one host species, the Siberian blue robin (*Larvivora cyane*), is a winter visitor to Malaysia (Robson 2008).

### Efficacy in Species Identification

The overall percentage of correct identification for the 95 COI barcodes based on BM and BCM was 96.84% and 86.31%, respectively. No sequences were reported as ambiguous for both BM and BCM. The incorrect identification rate was 3.15% for BM and 0% for BCM. The percentage of sequences without any match closer than the 3.00% threshold value for the BCM criteria was 13.68%.

### Pairwise Genetic Distance and DNA Barcode Gap

The pairwise intraspecific genetic divergence for the 8 nominal species from Malaysia sequences spanned from 0.00% to 17.64%. Minimal divergence was observed in *N. gallinarum*, *Parascoschoengastia heynemani* (Nadchatram & Upham, 1966), *Blankaartia acuscutellaris* (Walch, 1922), *Leptotrombidium imphalum* (Vercammen-Grandjean & Langston, 1976), and *Ascoschoengastia lorius* (Gunther, 1939). The highest intraspecific divergences of more than 10% were recorded for *Toritrombicula densipiliata* (Walch, 1922) (17.64%), *Odontacarus audyi* (Radford, 1946) (15.49%), and *Leptotrombidium deliense* (Walch, 1922) (11.63%) (Table 3). Furthermore, the study computed the minimum and maximum intraspecific genetic divergence across all 104 COI sequences. The intraspecific genetic divergence reflects variations in comparison to sequences obtained from GenBank. Specifically, the maximum intraspecific distance for *L. deliense* ranged from 11.63% within Malaysia to 32.40% when compared to sequences retrieved from GenBank, which originated from Thailand, Japan, Cambodia, China, and India (Table 3). Similar variations in genetic divergence were also seen in *B. acuscutellaris* and *L. imphalum*. Interspecific genetic divergence (Table 4) varied from 21.29% (lowest between *B. acuscutellaris* and *A. lorius*) to 45.00% (highest between *P. heynemani* and *L. deliense*).

**Table 3.** List of 24 chigger species and number of specimens with COI sequences (*N*) included for barcoding analysis, with minimum and maximum intraspecific genetic distances based on Kimura 2-parameter (K2P)

Tribe, subgenus	Species	*N* (this study)	Intraspecific genetic distance, % (maximum^a^)
Leeuwenhoekiini			
*Odontacarus*	***Odontacarus audyi***	(9)	0.00 to 15.49
	*O. scorpionivorus*	1	…
Schoengastiini			
*Ascoschoengastia*	*Ascoschoengastia* sp.	3	14.77 to 17.73
	***A. lorius***	(5)	0.00
	*A. indica*	4	1.25 to 18.75
*Neoschoengastia*	***Neoschoengastia gallinarum*** ^b^	16 (13)	0.0 to 7.93 (0.18^a^)
*Parascoschoengastia*	***Parascoschoengastia heynemani***	(5)	0.00 to 2.54
Trombiculini			
*Blankaartia*	***Blankaartia acuscutellaris*** ^b^	8 (4)	0.00 to 18.63 (0.36^a^)
*Leptotrombidium*	*Leptotrombidium akamushi*	1	…
	*L. chiangraiensis*	2	0.18
	***L. deliense*** ^b^	13 (4)	0.00 to 32.40 (11.63^a^)
	*L. fletcheri*	2	0.18
	***L. imphalum*** ^b^	7 (3)	0.18 to 21.97 (2.38^a^)
	*L. orientale*	1	…
	*L. pallidum*	3	0.90 to 1.64
	*L. intermedium*	1	…
	*L. palpale*	3	0.54 to 1.82
	*L. scutellare*	4	0.54 to 5.25
	*Leptotrombidium* sp.	1	…
*Ericotrombidium*	*Ericotrombidium kazeruni*	1	…
	*E. caucasicum*	1	…
*Neotrombicula*	*Neotrombicula gardellai*	1	…
	*N. vulgaris*	1	…
*Toritrombicula*	***Toritrombicula densipiliata***	(11)	0.00 to 17.64

*Note:* Eight species with newly generated sequences from this study are in bold. *N* = total number of sequences. The full matrix of intraspecific genetic distances is provided in the Supplementary Material.

^a^Maximum intraspecific genetic distance for newly generated sequences of Malaysian bird chiggers. Missing entries indicate that only one species of a particular genus was analyzed.

^b^Species with newly generated sequences and sequences retrieved from GenBank.

**Table 4.** Minimum interspecific genetic divergence calculated for COI sequences of eight chigger species based on Kimura 2-parameter (K2P)

	Interspecific pairwise genetic distance							
Species	1	2	3	4	5	6	7	8
*1. Neoschoengastia gallinarum*	0							
*2. Odontacarus audyi*	40.37	0						
*3. Parascoschoengastia heynemani*	38.76	33.65	0					
*4. Toritrombicula densipiliata*	27.42	38.4	32.98	0				
*5. Blankaartia acuscutellaris*	38.69	35.04	26.37	33.39	0			
*6. Ascoschoengastia lorius*	26.63	37.91	21.53	29.39	21.29	0		
*7. Leptotrombidium imphalum*	23.95	38.13	39.47	23.63	37.64	37.98	0	
*8. Leptotrombidium deliense*	35.48	35.98	42.24	25.45	32.75	34.38	22.72	0

**Fig. 3.**
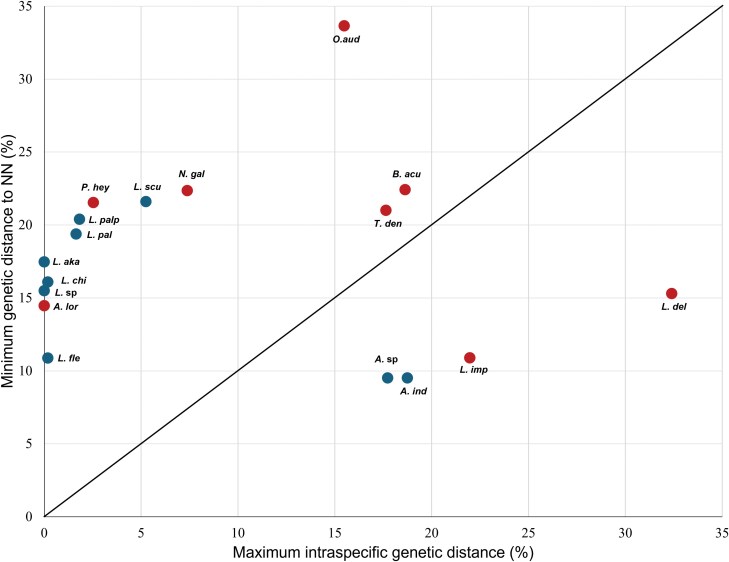
DNA barcoding gap represented by a scatterplot of maximum intraspecific genetic distance compared with the minimum genetic distance to NN of all 8 chigger species sequences from bird hosts in this study. Nodes above the 1:1 line indicate species with a barcode gap. The DNA barcode gap was present in 76.47% of all species examined. Species represented by a single sequence were excluded. Red dots include newly generated sequences (this study), whereas blue dots represent sequences from GenBank. O. aud = *Odontacarus audyi*; A. sp = *Ascoschoengastia* sp.; A. lor = *A. lorius*; B. acu = *Blankaartia acuscutellaris*; L. sp = *Leptotrombidium* sp.; L. del = *L. deliense*; L. imp = *L. imphalum*; L. aka = *L. akamushi*; L. pal = *L. pallidum*; L. palp = *L. palpale*; L. scu = *L. scutellare*; L. chi = *L. chiangraiensis*; L. fle = *L. fletcheri*; N. gal = *Neoschoengastia gallinarum*; P. hey = *Parascoschoengastia heynemani*; T. den = *Toritrombicula densipiliata*.

A scatterplot comparing intraspecific genetic distances and distances to the nearest neighbor (NN) revealed a DNA barcode gap in 76.47% of the species (Fig. 3). However, 4 species did not exhibit a distinct DNA barcode gap as evidenced by the overlap between the farthest conspecific distance and the distance to the NN. In the current study, *L. deliense* and *L. imphalum* did not exhibit a DNA barcode gap. Similarly, among the GenBank sequences, *Ascoschoengastia indica* (Hirst, 1915) also lacked a discernible DNA barcode gap.

### Phylogenetic Analyses Based on COI Sequences

**Fig. 4.**
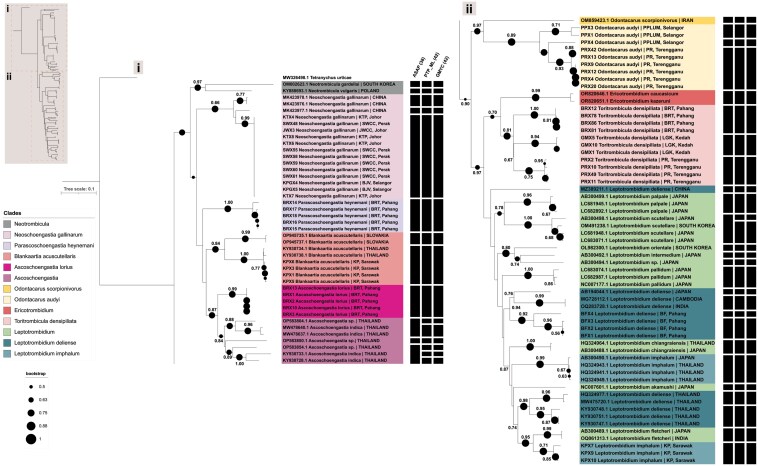
Phylogenetic tree of chiggers inferred through NJ and ML analysis based on 567 bp of mitochondrial COI sequences. The full view of the tree is shown in the top left corner, with the tree partitioned into 2 major clades: the upper clade labeled as i and the lower clade as ii. Bootstrap values are indicated by nodes on the branches, with bootstrap legend provided at the bottom left corner for NJ and on the branches for ML; Branch and node information are color-coded by trombiculid species and genus (for *Leptotrombidium* sp.). Sequences generated from the present study are indicated using sample ID as presented in Table 1. Vertical bars on the right are the OTUs generated by ASAP, PTP_ML and GMYC delimitation analyses. The tree is rooted with *Tetranychus urticae* (MW326498.1).

The phylogenetic analyses using NJ and ML methods displayed congruent tree topologies for COI sequences. All sequences generated in this study were newly produced and deposited into GenBank, representing the Malaysian region. In general, the COI sequences obtained from this study clustered within their respective genera as recognized in the current morphology-based taxonomy (*Ascoschoengastia*, *Blankaartia*, *Leptotrombidium*, *Neoschoengastia*, *Odontacarus*, *Parascoschoengastia*, and *Toritrombicula*) with robust bootstrap support in both trees, although the COI-based phylogeny was not always consistent with tribe-level classifications (Fig. 4). Thus, upon comparing these sequences to previously retrieved COI sequences from GenBank, 2 major clades were apparent. Notably, *B. acuscutellaris* (tribe: Trombiculini) was grouped among the Schoengastiini tribe (with *Ascoschoengastia*, *Parascoschoengastia*, and *Neoschoengastia*); whereas *O. audyi* (subfamily: Leeuwenhoekiinae) was clustered together with the remainder of the Trombiculini tribe (*Ericotrombidium*, *Leptotrombidium*, *Toritrombicula*).

While most genera constituted monophyletic groups, *T. densipiliata* and reference sequences for *Ericotrombidium* spp. formed a paraphyletic assemblage. Moreover, the sister relationships between *Ascoschoengastia*, *Parascoschoengastia*, and *Blankaartia* were poorly resolved (Fig. 4, upper clade [i]). Interestingly, however, both *O. audyi* and *T. densipiliata* exhibited evidence of intraspecific population structure linked to sampling location. For *Leptotrombidium* spp., for which the greatest number of reference sequences were available, several inconsistencies in species placements were observed in the phylogeny. The *L. imphalum* and *L. deliense* data from the present study did not fall into the same clades as putatively conspecific sequences from neighboring Thailand. Rather, *L. deliense* from Malaysia appeared to be more closely related to conspecifics from India, Cambodia, and Japan; whereas *L. imphalum* from Malaysia formed a clade with *Leptotrombidium fletcheri* (Womersley & Heaslip, 1943).

### Species Delimitation Analysis

The number of OTUs identified through species delimitation analysis varied slightly depending on the method used. The ASAP method revealed 38 OTUs, while both the PTP_ML and GMYC methods identified 42 OTUs for all 54 sequences from this study (eight nominal species) and 50 GenBank sequences (Fig. 4). In the ASAP analysis, the first partition with the lowest ASAP score (5.00), giving a threshold value of 0.1077, was chosen from the 10 best partitions found to be congruent with the nominal species from this study. Meanwhile, both the PTP_ML and GMYC methods delimited OTUs that shared the same assignations. Overall, all 3 species delimitation analyses exhibited agreement in the classification of the 8 nominal species from Malaysia. Among these, *N. gallinarum*, *A. lorius*, *P. heynemani*, and *B. acuscutellaris* were treated as single taxonomic units, with their members considered inseparable. Specifically, *B. acuscutellaris* individuals from Malaysia were identified as the same taxonomic entity as those from Thailand. Conversely, *L. imphalum* from Malaysia was also identified as a single OTU, but distinct from those found in Thailand and Japan. Notably, *O. audyi* and *T. densipiliata* contained more than one OTU by species delimitation methods, corresponding with their respective sites of collection (*O. audyi*—PPLUM and PR; *T. densipiliata*—BRT, LGK, and PR), although both formed monophyletic clades with robust bootstrap support based on nominal species designation. Meanwhile, *L. deliense* was split into 2 distinct entities by all 3 delimitation analyses, despite being collected from the same site and bird host species.

## Discussion

The mitochondrial-encoded COI gene marker has previously been shown to be amplified successfully across a wide diversity of invertebrates, including trombiculid mites (Dabert et al. 2008, Doña et al. 2015, Antonovskaia 2018, Kumlert et al. 2018), using universal primers. The joint use of morphological identification with COI barcoding, alongside multiple DNA-based species delimitation methods (Pons et al. 2006, Fujisawa and Barraclough 2013, Zhang et al. 2013, Puillandre et al. 2021), will not only improve the accuracy of specimen assignment but also contribute to the expansion of a reference database for this marker (Carugati et al. 2022). This supports BLAST searches for efficient molecular identification and could help unravel associations between chigger species, their hosts, endosymbionts, and pathogens, particularly in the context of scrub typhus transmission (Kumlert et al. 2018). In this study, COI sequences were obtained from 8 species of Malaysian trombiculid mites and assessed for the first time using DNA barcoding.

The COI gene marker effectively matched 86% and 96% of the species with corresponding morphological identifications based on the BCM and BM approaches, respectively. However, there were exceptions, with certain species being incorrectly identified according to BM due to either overlapping genetic distances with their NN species or high intraspecific distances (Izwan-Anas et al. 2023). These discrepancies were associated with species recovered as nonmonophyletic in the phylogenetic analyses. The term “DNA barcode gap” refers to the disparity in genetic distances between the closest conspecific individuals and the NN species in DNA barcode sequences (Robinson et al. 2009). The observation of a ~76% DNA barcode gap in this study indicates a clear separation between the farthest conspecific distance and the NN distance. According to Meyer and Paulay (2005), a larger barcode gap signifies a distinct delineation between species, leading to more accurate species identification using DNA barcoding methods. In this study, the majority of species displayed a DNA barcode gap and demonstrated monophyly within their respective species and genera, with the exception of the genus *Leptotrombidium*, since *L. deliense* and *L. imphalum* did not display a DNA barcode gap. In certain instances, genetic overlap may arise from species having large genetic diversity (DeSalle et al. 2005).

Additionally, other potential factors that might contribute to paraphyly among *Leptotrombidium* spp. include inadequate phylogenetic signal, imperfect taxonomy, interspecific hybridization, incomplete lineage sorting, and paralogs for marker loci (Kadosaka et al. 1994, Funk and Omland 2003). Furthermore, nuclear mitochondrial pseudogenes (*numts*) can lead to paralogous sequences and ambiguity in DNA barcoding (Song et al. 2008). A study by Shao et al. (2005) also identified a pseudogene for small subunit rRNA (PrrnS) in one of the 4 *Leptotrombidium* spp. studied, specifically in the mitogenome of *L. pallidum*. Our study confirmed the absence of *numts* in the COI barcode sequences generated for Malaysian chiggers. Thus, the potential sequence ambiguity resulting from *numts* during DNA barcoding was reduced.

The current study revealed a notable degree of genetic divergence of COI gene sequences among individuals of 8 identified species of Malaysian bird-associated chiggers, with divergence values ranging from 0.00% to 17.64%. Generally, a genetic divergence of less than 2% to 3% is considered indicative of individuals belonging to the same species (Hebert et al. 2003, Lee and Choi 2022), whereas a divergence exceeding 3% represents a substantiated threshold, indicating a distinct separation between sister phylogroups (Rivera and Currie 2009, Pramual and Adler 2014, Juan et al. 2015, Pramual et al. 2016, Klimov et al. 2019, Zajkowska et al. 2023). Among the chigger species studied—*A. lorius*, *N. gallinarum*, and *P. heynemani* (tribe Schoengastiini), as well as *B. acuscutellaris and L. imphalum* (tribe Trombiculini)—a maximum intraspecific variation of 2.54% was observed, indicating these species form genetically cohesive groups consistent with their morphological classification. Accordingly, several studies have revealed that the maximum range of intraspecific variation observed in DNA barcoding studies of insects and other invertebrates typically falls between 3% and 3.9% (Ball et al. 2005, Cywinska et al. 2006, Smith et al. 2006, Carew et al. 2007). However, in the case of mites, the range of intraspecific COI divergence may exceed this range in some instances. For instance, Klimov et al. (2019) reported high COI divergence between 2 lineages of the scab mite *Caparinia* (7.4% to 7.8%) and 4.2% between sympatric lineages of the house dust mite *Dermatophagoides farinae*, while a within-species divergence of 4.3% was observed in the mold mite *Tyrophagus putrescentiae* (Murillo et al. 2018).

Remarkably, the nominal species of *B. acuscutellaris*, *N. gallinarum*, and *L. imphalum* demonstrated larger divergence when compared to the publicly available COI gene sequences from other countries and this was further supported by all 3 species’ delimitation analyses, resulting in different OTUs within these nominal species. Fragmented distributions caused by landscape topography, along with biological and oceanographic limitations on dispersal range, can induce isolation and founder effects between population groups. Over time, these factors can inhibit genetic exchange, resulting in measurable genetic differentiation driven by mutation, natural selection, and genetic drift (Riginos and Nachman 2001; Hazlitt et al. 2006). The extent to which geographic isolation drives population differentiation within trombiculid species has received little attention to date outside *N. gallinarum* (Rajasegaran et al. 2024), but it is noteworthy that this species and *L. imphalum* have been reported from multiple Asian countries. Moreover, *L. deliense* is distributed across the Indian subcontinent, China, Southeast Asia, New Guinea, and tropical Australia; while *B. acuscutellaris* is a species known not only from Asia, but also Africa and Europe (Stekolnikov 2021). It has been speculated that bird migrations have enabled these vast distributions of many trombiculid species in the absence of intrinsic dispersal ability (Kim et al. 2017).

The classification of the genus *Toritrombicula* (Sasa, 1954) is intricate due to uncertain authorship, attributed to various sources: Sasa et al. (1953), Sasa (1954), and others (Vercammen-Grandjean 1960, Loomis 1956; Vercammen-Grandjean and Langston 1976, Takahashi et al. 2012). Within this genus, *T. densipiliata* was discovered across 3 sites in Malaysia and associated with 8 bird host species (Koosakulnirand et al. 2023), including 2 migratory bird species (*Cyornis brunneatus* and *L. cyane*). Notably, *T. densipiliata* exhibited the highest observed intraspecific genetic distance. According to Kreipe et al. (2015), while delimiting species using the COI fragment in certain acarine groups is feasible, its accuracy can be influenced by geographic variability. Delimitation analyses on *T. densipiliata* revealed distinct entities based on their geographical locations. The maximum genetic divergence was observed between the mainland (BRT) and island (GMX) populations. Therefore, the substantial genetic divergence between these 2 populations could have resulted from geographic isolation and restricted genetic interchange with neighboring mainland or island biota (Kier et al. 2009).

However, the intraspecific genetic distance not only highlighted distinct divergence among multiple, separated populations but also within a population of a species from a single geographic location. For instance, a maximum difference of 15.49% was observed between an individual chigger designated as PPX1 (host—*Cacomantis sepulcralis*, nonmigratory) and an individual designated as PPX4 (host—*Copsychus malabaricus,* nonmigratory), both of which were nominally assigned as *O. audyi*. Moreover, despite being clustered in a monophyletic group, delimitation analysis split these individuals into separate entities. Conversely, the *O. audyi* population from PR, Terengganu, was identified as a single entity despite being collected from different bird hosts, whereas 2 distinct entities were obtained for the population from PPLUM, each corresponding to specific hosts. This indicates the possibility of host-specific adaptation, where chigger species with a strong preference for avian hosts may have genetically diverged in response to bird species’ ecological niches. However, larger chigger sample sizes from a greater variety of hosts, ideally from a single geographic region to reduce the impact of environmental factors, would be required to test this hypothesis effectively.

The intraspecific genetic distance for *L. deliense* in Malaysia was found to be 11.63%, which significantly increased when comparing with the COI sequences obtained from GenBank, showing nearly 3 times greater divergence. Two distinct entities were generated by delimitation analysis for this species, despite being collected from a single location and nonmigratory host, demonstrating evidence of genetic polymorphism. The observed genetic diversity in this species could be attributed to their natural transient carriage on bird hosts during feeding, facilitating interactions between mite populations within or between microhabitats, potentially resulting in breeding among genetically distinct groups despite partial reproductive isolation (Kumlert et al. 2018). Overall, *L. deliense* exhibited marked polyphyly, which resulted in a total of 7 OTUs across 6 countries included in this study. The highest divergence was observed against the population from Malaysia, particularly when compared with a single COI sequence obtained from China. Notably, despite being designated as distinct entities, the Malaysian *L. deliense* specimens collected from bird hosts (*Pellorneum ruficeps*) were clustered together with individual sequences from Japan, Cambodia, and India, but not with the population from Thailand, although the latter shares a land border with Malaysia as a neighboring country. However, accurately measuring the genetic variation between these populations is currently challenging due to the scarcity of available sequences from Cambodia, China, and India, along with their unpublished status. These findings suggest that *L. deliense* may actually represent a species complex, though further research is needed to clarify these observations, as analyses based on a single mitochondrial locus could be biased due to prior population bottlenecks or selective sweeps (Shtolz and Mishmar 2019).

Among trombiculids, the genus *Leptotrombidium* presents some of the most significant taxonomic challenges due to its great diversity and morphological variation (Vercammen-Grandjean and Langston 1976). The species in this genus are well known to infest a wide range of hosts and at the same time to harbor the causative agent of scrub typhus disease, *O. tsutsugamushi* (Traub and Wisseman 1968, Stekolnikov 2013, Santibáñez et al. 2015). As the main scrub typhus vector in Asia, *L. deliense* is a generalist species with a wide geographic distribution and host preferences, which could potentially lead to the evolution of a species complex. In comparing our current study with previous research, we observed parallel findings regarding this genus. A study by Kumlert et al. (2018) reported that sequences of *L. deliense* from Lao PDR formed a distinct clade separated from previously recorded sequences from northern Thailand. Moreover, for other *Leptotrombidium* spp., Lee and Choi (2022) recovered 2 genotypes in *L. pallidum* and *L. palpale*, and 3 genotypes in *L. scutellare* using the COI gene marker. An intraspecific distance of 10.82% with 57 variable sites was reported by these authors for *L. pallidum* genotypes A and B. Additionally, a total of 142 variable sites with genetic distances ranging from 12.74% to 17.07% were recovered in *L. scutellare* from the single region studied (Lee and Choi 2022). However, these authors reported that the ITS2 sequence was identical within all 3 *Leptotrombidium* spp., providing a convenient locus for species identification. Hence, further research utilizing the nuclear-encoded ITS2 marker is needed to validate the *Leptotrombidium* spp. examined within the current study.

The notable intraspecific variation observed in this study underscores the need for investigations into potential cryptic species in multiple trombiculid genera. A comprehensive sample size from diverse localities would enhance the reliability of molecular barcoding approaches and is essential when many chigger species have distributions spanning multiple countries (Meyer and Paulay 2005). In certain taxa, the COI region alone may be insufficient for assessing species boundaries due to its rapid evolutionary rate, maternal inheritance, and associated genetic variation, which is particularly pronounced in arthropods (Kanduma et al. 2016, Zhou et al. 2020 , Lee and Choi 2022, Rajasegaran et al. 2024). Therefore, a multilocus sequence approach that targets both the mitochondrial and nuclear genomes could enhance the accuracy of species identification by addressing the limitations of a single marker (Gadagkar et al. 2005, Rajasegaran et al. 2024). Since chiggers are known to host various vertically transmitted bacteria capable of inducing reproductive manipulations (Chaisiri et al. 2023) and cytonuclear discordance (Cariou et al. 2017), it is crucial to explore potential cryptic species using nuclear genome fragments such as the 18S and 28S rRNA genes and ITS2, not only mitochondrial markers (Antonovskaia 2018, Rajasegaran et al. 2024).

## Conclusion

In combination with morphology-based taxonomic descriptions (Koosakulnirand et al. 2023), this study has generated DNA barcodes for *A. lorius*, *B. acuscutellaris*, *L. deliense*, *L. imphalum*, *N. gallinarum*, *O. audyi*, *P. heynemani*, and *T. densipiliata*, contributing to the establishment of a sequence library of bird-associated trombiculids in Malaysia. The COI sequences derived from this study for *A. lorius*, *P. heynemani*, *O. audyi*, and *T. densipiliata*, mark their first reference barcodes to be archived in the NCBI nucleotide database. The DNA barcoding approach has effectively differentiated most taxonomically well-defined species while simultaneously revealing cryptic diversity.

## Supplementary material

Supplementary material is available at *Journal of Medical Entomology* online.

tjaf078_suppl_Supplementary_Materials

## Data Availability

The COI sequences generated were deposited in the GenBank database under the accession numbers PP739731-PP739779, and PP741800.
